# The significance of preoperative estimated glomerular filtration rate on survival outcomes in patients who underwent radical cystectomy and non-continent urinary diversion

**DOI:** 10.1590/S1677-5538.IBJU.2019.0205

**Published:** 2020-03-20

**Authors:** Ertugrul Sefik, Serdar Celik, Bulent Gunlusoy, Ismail Basmaci, Ibrahim H. Bozkurt, Tansu Degirmenci

**Affiliations:** 1 Department of Urology Bozyaka Training and Research Hospital Izmir Turkey Department of Urology, Bozyaka Training and Research Hospital, Izmir, Turkey

**Keywords:** Urinary Bladder Neoplasms, Cystectomy, Urinary Diversion

## Abstract

**Purpose:**

To evaluate the influence of preoperative renal function on survival outcomes in patients who underwent radical cystectomy (RC) with non-continent urinary diversion (UD).

**Materials and Methods:**

A total of 132 patients with bladder cancer who underwent RC with non-continent UD due to urothelial carcinoma from January 2006 toMarch 2017 at our tertiary referral center were retrospectively evaluated. Patients were divided into 2 groups as those with estimated glomerular filtration rate (eGFR) <60mL/min/1.73 m2 and ≥60mL/min/1.73 m2 according to preoperative eGFR levels. Patients’ characteristics, preoperative clinical data, operative data, pathologic data, oncologic data and complications were compared between the groups.

**Results:**

The mean age was 64.5±8.7 (range: 32 - 83) years and the median follow-up was 30.9±31.7 (range: 1-113) months. There were 46 patients in Group 1 and 86 patients in Group 2. There was no difference in cancer-specific mortality (45.6% for group 1 and 30.2% for group 2, p=0.078) and survival (56.8±8.3 months for group 1 and 70.5±5.9 months for group 2, p=0.087) between the groups. Overall mortality was higher (63% for group 1 and 40.7% for group 2, p=0.014) and overall survival (43.6±6.9 months for group 1 and 62.2±5.8 months for group 2, p=0.03) was lower in Group 1 compared to Group 2.

**Conclusions:**

Overall mortality was higher and overall survival was lower in patients with preoperative eGFR <60mL/s. More patients had preoperative hydronephrosis with eGFR< 60mL/s.

## INTRODUCTION

Radical cystectomy (RC) with extended pelvic lymph node dissection is the best choice of treatment in patients with non-metastatic muscle-invasive and high-risk non-muscle invasive bladder cancer ( 1 - 4 ). The procedure is completed with urinary diversion (UD) after the removal of the bladder. RC with UD is a 2-step, complex surgical procedure and is associated with significant risks of perioperative and long-term morbidity and mortality, including renal function deterioration and development of chronic renal disease (CKD) ( 5 , 6 ).

The etiology of a renal function decrease after RC is likely multifactorial, including age-related changes, potential nephrotoxic chemotherapy, and the impact of patient comorbidities, which are frequent in such a population, and postoperative urinary tract obstruction and infection-related complications ( 7 ). Renal dysfunction is fairly common in this group of patients. Patients with bladder cancer largely comprise middle aged and elderly people ( 8 ). This is indicative of the presence of many morbidities that accompany bladder cancer in patients. Hamano et al. found that advanced preoperative CKD stage was significantly associated with poor oncological outcomes of bladder cancer after RC ( 8 ).

Comorbidities such as hypertension (HT), diabetes mellitus (DM) and vascular disease are important risk factors for the development of CKD at advanced age ( 9 ). Matsumoto et al. discussed the precise biological mechanism of association between tumor aggressiveness and CKD status with possible explanations. They found chronic inflammation induced by continuous exposure to oxidative stress and accompanying immune deficiency to be responsible mechanisms for CKD ( 10 ).

An important point in the evaluation of renal dysfunction is the method of choice to calculate the renal function. Most studies evaluate renal function variations using serum Δ creatinine as a surrogate value for the estimated glomerular filtration rate (eGFR) ( 11 ). Makino et al. assessed eGFR alterations over the years and risk factors for decreasing eGFR. Deterioration in renal function in early and late postoperative years was defined as a ≥25% decrease in the eGFR from preoperative to postoperative year one and a reduction in the eGFR of >1mL/min/1.73m2 annually in subsequent years ( 12 ).

In this study, we aimed to evaluate the influence of preoperative renal function on oncological outcomes and prognosis in patients who underwent RC and non-continent UD.

## MATERIALS AND METHODS

A total of 132 patients with bladder cancer who underwent RC with non-continent UD due to urothelial carcinoma from January 2006 to March 2017 at our tertiary referral center were retrospectively evaluated. Patients were divided into 2 groups as eGFR <60mL/s and ≥60mL/s according to preoperative eGFR levels. Patients without urothelial carcinoma on pathological examination, presence of upper tract urothelial carcinoma or obstructive stones and patients with incomplete medical records were excluded from the study. Patient characteristics, preoperative, operative and follow-up data were reviewed. The indications for RC were tumor invasion into the muscularis propria or prostatic stroma, or non-muscle-invasive disease (Ta, T1, or carcinoma in situ) refractory to transurethral resection with intravesical therapy.

Patient’s characteristics (age, gender, presence of DM, HT and other comorbidities), preoperative clinical data (preoperative and postoperative at 3 months and creatinine and eGFR levels, American Society of Anesthesiologists (ASA) score, Eastern Cooperative Oncology Group (ECOG) performance score, Charlson comorbidity index and hydronephrosis presence, grade and laterality), operative data (operation time and diversion type data), pathologic data (preoperative T stage, tumor grade and carcinoma in situ (CIS) presence, postoperative T stage and tumor grade, surgical margin positivity, number of dissected lymph nodes, positive lymph node ratio, lymph node metastasis and percentage of positive lymph node data), oncologic data (upstaging, adjuvant chemotherapy, overall mortality (OM) and overall survival (OS), cancer specific mortality (CSM) and CSS and complications (hospitalization time, early medical complication, early surgical complications, complication data of Clavien-Dindo classification) were evaluated.

Type of incontinent urinary diversions were ureterocutaneostomy and incontinent ileal conduit. Creatinine was defined as difference between postoperative 3rd month creatinine and preoperative creatinine. Hydronephrosis was defined by anteroposterior diameter of the renal pelvis >10mm which was diagnosed by renal ultrasound or CT scan with or without secondary changes of renal parenchyma or renal function.

### Statistical analysis

Data were analyzed using the Statistical Package for Social Sciences, version 20.0 (SPSS, Chicago, Ill) software program. According to preoperative eGFR levels, patients were divided into two groups as preoperative eGFR <60mL/s (Group 1) and preoperative eGFR ≥60mL/s (Group 2) groups. Mann-Whitney U test and Pearson Chi-square test analyses for univariate analysis and binary logistic regression analysis for multivariate analysis were used between the groups. In addition, Kaplan-Maier survival analysis and the log-rank test were used for OS and CSS times between groups. In addition, same tests were used for univariate and multivariate analysis of the factors affecting on overall and cancer specific deaths. A Cox regression model was created for evaluating the predictive factors on overall survivals. Data are given as mean±SD. However, results of analysis are given as median data. Statistical significance was defined as p <0.05.

## RESULTS

### Patients characteristics

The mean age was 64.5±8.7 (range: 32-83) years and the median follow-up time was 30.9±31.7 (range: 1-113) months. Consistent with previous data, there was a limited number of female patients (12 of 132, 9%). Mean OS and CSS of all patients were 56.3±4.7 and 67.1±5 months, respectively. There were 46 patients in Group 1 and 86 patients in Group 2. Comparison of patient’s characteristics and preoperative clinical data between Group 1 and Group 2 according to preoperative eGFR levels were given in Table-1 . In univariate analysis, the distributions of HT, DM, comorbidity data, ASA score, ECOG performance score and Charlson comorbidity index were similar, only preoperative hydronephrosis presence and hydronephrosis laterality were found to be significantly higher in Group 1 compared to Group 2. Preoperative and postoperative creatinine and eGFR at the third month and creatinine levels of the groups are given in Table-1 to show mean creatinine and eGFR data. When we evaluated the peroperative and postoperative results and pathologic data between the groups, any prognostic and pathologic data were significant.

**Table 1:** Comparison of patient’s characteristics and preoperative findings between eGFR <60mL/min/1.73m2 and eGFR ≥60mL/min/1.73m2 groups according to preoperative eGFR levels.

		Preoperative eGFR <60mL/min/1.73m2 (n=46)	Preoperative eGFR ≥60mL/min/1.73m2 (n=86)	p
Age (years) (mean±SD)		65.9±9.5	63.7±8.2	0.145
**Gender**	Female	7	5	
	Male	39	81	
Preoperative creatinine (mean±SD)		1.69±0.57	0.96±0.15	-
Preoperative eGFR (mean±SD)		41.6±13	80±16.9	-
Postoperative 3 month creatinine (mean±SD)		1.72±0.66	1.21±0.57	<0.001
Postoperative 3 month eGFR (mean±SD)		45±18.4	69.9±22.9	<0.001
Δ creatinine (mean±SD)		0.04±0.66	0.26±0.55	0.015
**ASA**	1	1	4	0.218
	2	3	46	
	3	12	34	
	4	0	2	
**ECOG Performance score**	0	8	25	0.364
	1	24	36	
	2	9	16	
	3	3	7	
	4	1	0	
**Charlson comorbidity index**	0	0	1	0.295
	1	1	2	
	2	13	13	
	3+	32	70	
DM, n (%)		10 (21.7)	15 (17.4)	0.548
HT, n (%)		24 (52.2)	30 (34.9)	0.054
Any comorbidity, n (%)		37 (80.4)	60 (69.8)	0.186
**Preoperative hydronephrosis**	positive	30	23	<0.001
	negative	16	63	*<0.001
**Hydronephrosis laterality**	unilateral	18	21	0.01
	bilateral	12	2	*0.019
**Preoperative hydrophrosis grade**	1	1	4	0.276
	2	9	4	
	3	15	10	
	4	5	5	
Preoperative nephrostomy tube insertion for grade 3-4 hydrophrosis		12 (60)	7 (46.7)	0.767

Mann Whitney U test and Pearson Chi-square test.

*Binary logistic regression analysis for significant data of univariate analysis results.

**ASA** = American Society of Anesthesiologists; **ECOG** = Eastern Cooperative Oncology Group; **DM** = Diabetes mellitus; **HT** = Hypertension

### Oncological outcomes

**Figure 1:**
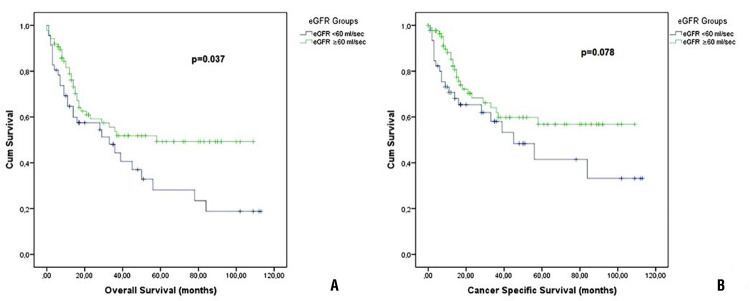
A) Overall survival plots of Kaplan-Maier analysis. B) Cancer specific survival plots of Kaplan-Maier analysis.

Mean OS and CSS of all patients were 56.3±4.7 and 67.1±5 months, respectively. Overall and cancer specific deaths were 64 and 47 in all patients. In the comparison of oncological outcomes, although there was no difference in cancer specific mortality (45.6% for group 1 and 30.2% for group 2, p=0.078) and CSS (56.8±8.3 months for group 1 and 70.5±5.9 months for group 2, p=0.087) between the groups, OM was higher (63% for group 1 and 40.7% for group 2, p=0.014) and OS (43.6±6.9 months for group 1 and 62.2±5.8 months for group 2, p=0.03) was lower in Group 1 compared to Group 2. Survival plots are given in Figure-1 . Furthermore, upstaging and adjuvant chemotherapy rates were similar between the groups. Oncological data and survival findings are given in Table-2 and Table-3 . Also, univariate and multivariate analysis results of the factors affecting on overall and cancer specific deaths are given in Table-4 . Preoperative eGFR was significantly associated with overall death. In addition, preoperative eGFR (p=0.041, OR:0.514, CI:1.022-2.738), preoperative hydronephrosis (p=0.002, OR:0.878, CI:0.240-0.721), age (p=0.038, OR:0.03, CI:1.002-1.061) and pathological T stage (p=0.013, OR:0.349, CI:0.136-0.619) were found to be associated with overall survival after radical cystectomy in Cox regression model (p=0.001). In groups, hospitalization time, early medical and surgical complication rates, and complication rates according to Clavien-Dindo classification were also similar.

**Table 2:** Comparison of operative and pathologic data between Group 1 and Group 2.

		Preoperative eGFR <60mL/min/1.73 m2 (n=46)	Preoperative eGFR ≥60mL/min/1.73 m2 (n=86)	P*
**Preoperative T stage**	≤T1	6	9	0.721
	T2	38	75	
	T3	2	2	
**Preoperative tumor grade**	Grade1	1	2	0.485
	Grade2	3	2	
	Grade3	42	82	
**CIS**	positive	10	30	0.108
	negative	36	55	
Operation time (hours)		5.6±1.3	5.8±1.1	0.369
**Postoperative T stage**	T1	8	20	0.364
	T2	17	39	
	T3	8	13	
	T4	13	14	
**Postoperative tumor Grade**	1	3	5	0.905
	2	1	3	
	3	37	68	
**Surgical margin positivity**	positive	13	13	0.07
	negative	33	73	
Number of dissected lymph node		12.4±5.9	13.2±4.9	0.430
Positive lymph node ratio		1.1±2.4	0.4±1.2	0.121
**Lymph node metastasis**	Positive	11	14	0.318
	Negative	34	68	
Percentage of positive lymph node		8.9±19.1	3.5±9.4	0.136
**Diversion type**	Ureterocutaneostomy	23	32	0.156
	Ileal conduit	23	54	

*Mann Whitney U test and Pearson Chi-square test

**CIS** = Carcinoma In Situ

**Table 3:** Comparison of postoperative data, complications and survival findings between Group 1 and Group 2.

		Preoperative eGFR <60mL/min/1.73 m2 (n=46)	Preoperative eGFR ≥60mL/min/1.73 m2 (n=86)	p
**Upstaging**	Positive	22	55	0.073
	Negative	24	31	
**Upstaging**	upstaging	24	31	0.160
	downstaging	5	17	
	No differance	17	38	
Adjuvant chemotherapy, n (%)		12 (26.1)	22 (25.6)	0.950
Overall Mortality, n (%)		29 (63)	35 (40.7)	0.014
				*0.015
Overall Survival		43.6±6.9	62.2±5.8	#0.037
Cancer Specific Mortality, n (%)		21 (45.6)	26 (30.2)	0.078
Cancer Specific Survival		56.8±8.3	70.5±5.9	#0.087
Surgery time (hours)		5.6±1.3	5.8±1.1	0.518
Hospitalization time		11.3±4.6	12.1±6.3	0.475
**Early medical complication**	Positive	14	20	0.369
	Negative	31	66	
**Early surgical complication**	Positive	15	41	0.095
	Negative	31	45	
**Clavien-Dindo**	1	5	8	0.357
	2	34	51	
	3a	1	2	
	3b	3	15	
	4a	1	7	
	5	2	3	

Mann Whitney U test and Pearson Chi-square test

*Binary logistic regression analysis for significant data of univariate analysis results

# Kaplan-Maier survival analysis and the log-rank test

**Table 4:** Univariate and multivariate analysis of the factors affecting on overall and cancer specific death.

		Overall death			Cancer specific death		
	
		n=64	p	p*	n=47	p	p*
**Preoperative hydronephrosis**	Positive	35	0.001	0.071	27	0.002	0.052
	Negative	29			20		
**Preoperative T stage**	≤T1	8	0.501	-	5	0.247	-
	T2	53			39		
	T3	3			3		
**eGFR (mL/sec)**	<60	29	0.014	p=0.040	21	0.078	-
	≥60	35		HR:2.33 (CI:1.04-5.22)	26		
**Postoperative T stage**	T1	10	<0.001	0.118	6	<0.001	0.614
	T2	19			13		
	T3	13			10		
	T4	22			18		
**Postoperative tumor Grade**	1	4	0.988	-	3	0.890	-
	2	2			2		
	3	55			40		
**Surgical margin positivity**	Positive	22	<0.001	0.087	19	<0.001	0.109
	Negative	42			28		
**Lymph node metastasis**	Positive	17	0.016	0.284	15	0.003	0.076
	Negative	42			28		
**Upstaging**	Positive	36	0.001	0.544	29	0.001	0.467
	Negative	28			18		

*Multivariate analysis results

## DISCUSSION

There is ongoing debate about the effect of preoperative patient status on the surgical outcomes after radical cystectomy. CKD, HT, DM and vascular diseases are well-known risk factors which have negative impact on surgical outcomes. An independent, graded association was observed between reduced eGFR and the risk of death, in a large, community-based population. These findings highlight the clinical and public health importance of chronic renal insufficiency ( 13 ). Interest in the influence of preoperative renal insufficiency on cancer prognosis has increased because of its prevalence in elderly patients with muscle-invasive bladder cancer ( 3 ). Eisenberg et al. reported that decreased renal function is noted in most patients during long-term follow-up after radical cystectomy and approximately 70% of patients undergoing RC with UD experience eGFR decline postoperatively. They also stated that choice of urinary diversion was not independently associated with decreased renal function ( 7 ).

Despite the variety of diversion techniques, either continent or non-continent, patients undergoing RC have a life-long risk of CKD ( 11 ). Continent diversion methods are mostly not preferred in the case of preoperative CKD, while de Toledo et al. emphasized that gastric neobladder can be used in highly selected cases (e.g., renal insufficiency) because of its high morbidity and mortality rates ( 14 ). According to the selected diversion method, our study group consisted of patients with non-continent diversion. There were 77 patients with ileal conduit diversion (ICD) and 55 patients with ureterocutaneostomy (UC). Our patients were divided into 2 groups to compare the effect of preoperative eGFR on oncologic results. In patients with preoperative eGFR <60mL/s, preoperative creatinine level increased from 1.69±0.57 to 1.72±0.66. However, creatinine levels were worse in patients with preoperative eGFR ≥60mL/s (increased from 0.96±0.15 to 1.21±0.579). This situation can be explained as relative improvement due to the regression of preoperative hydronephrosis in patients with preoperative eGFR <60mL/s. In the comparison of groups, more patients already had preoperative hydronephrosis in Group 1. Urinary tract obstruction was the leading cause of long-term renal function impairment, regardless of whether the patient had ileal conduit diversion or orthotopic ileal bladder substitution. Also, Eisenberg et al. reported that age, preoperative kidney function and chronic hypertension, and the postoperative complications of hydronephrosis, pyelonephritis and uretero-enteric anastomotic stricture were associated with an increased risk of decreased renal function ( 7 ). Our findings support the effect of preoperative hydronephrosis on renal functions and oncologic outcomes.

In recent years, population-based studies reported a slow increase in cancer risk as CKD status progressed ( 15 , 16 ). In a previous study, it was found that patients with CKD had worse prognosis, higher tumor recurrence and progression rates in primary non-muscle invasive bladder cancer ( 17 ). In some other studies, it was shown that poor oncologic results accompany CKD in muscle-invasive bladder cancer patients who underwent RC ( 18 , 19 ). There is an age-dependent physiological decrease in eGFR, which was defined as a >10mL/min/1.73m2 drop in eGFR from baseline, which occurs per decade ( 20 ). In the current study, overall mortality and survival were significantly worse with preoperative eGFR <60mL/s. CKD might not only limit long-term outcomes by increasing the risk of cardiovascular morbidity and mortality, but also compromise short-term outcomes ( 13 ).

Long-term renal function after RC can be adversely affected by several factors, including age, potential nephrotoxic chemotherapy, comorbidities, and diversion-related factors ( 11 ). Physicians dealing with uro-oncology mostly prefer non-continent diversion techniques instead of orthotopic neo-bladder formation in patients with concomitant morbidities such as CKD, cardiovascular or advanced chronic obstructive lung disease.

We showed that overall survival and mortality were poorly affected by low eGFR in patients undergoing non-continent diversion. Our study was limited by its retrospective design and small number of patients. Also, the threshold value to define renal failure is heterogeneous in different studies. Blood urea and creatinine estimations are easy and inexpensive, but these biochemical parameters can be affected by different metabolic events. In fact, each diversion method leads to subtle metabolic changes causing confused results. As far as finding the ideal method, eGFR seems to give best results for the measurement of renal failure.

## CONCLUSIONS

Preoperative hydronephrosis, which is a well-known prognostic factor in patients undergoing radical cystectomy, was significantly higher in patients with eGFR <60mL/s. Overall mortality was higher and overall survival was lower in patients with preoperative eGFR <60mL/s. Renal dysfunction is an important risk factor for overall survival in patients who undergo radical cystectomy.
